# The Evolution of Insulin Administration in Type 1 Diabetes

**DOI:** 10.4236/jdm.2021.115021

**Published:** 2021-11-17

**Authors:** Catherina T. Pinnaro, Michael J. Tansey

**Affiliations:** 1 University of Iowa Stead Family Department of Pediatrics; 2 Fraternal Order of Eagles Diabetes Research Center

**Keywords:** Type 1 diabetes (T1D), insulin, hybrid-closed loop, hypoglycemia

## Abstract

Insulin has been utilized in the treatment of type 1 diabetes (T1D) for 100 years. While there is still no cure for T1D, insulin administration has undergone a remarkable evolution which has contributed to improvements in quality of life and life expectancy in individuals with T1D. The advent of faster-acting and longer-acting insulins allowed for the implementation of insulin regimens more closely resembling normal insulin physiology. These improvements afforded better glycemic control, which is crucial for limiting microvascular complications and improving T1D outcomes. Suspension of insulin delivery in response to actual and forecasted hypoglycemia has improved quality of life and mitigated hypoglycemia without compromising glycemic control. Advances in continuous glucose monitoring (CGM) and insulin pumps, efforts to model glucose and insulin kinetics, and the application of control theory to T1D have made the automation of insulin delivery a reality. This review will summarize the past, present, and future of insulin administration in T1D.

## 1. Introduction

**Figure 1.**
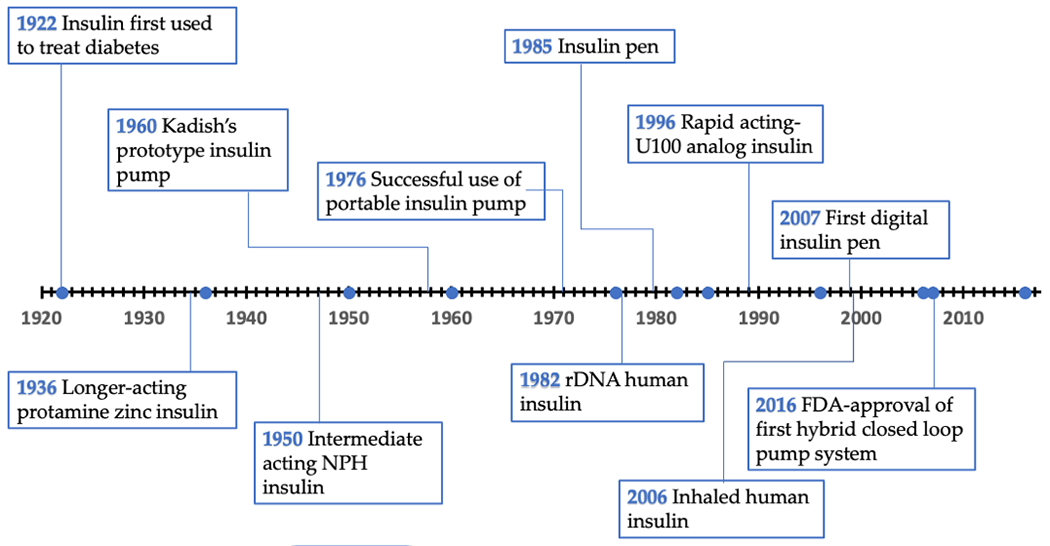
Timeline of important advancements in insulin delivery

The discovery, purification, and subsequent demonstration that insulin administration could reduce blood and urine glucose levels and reverse ketoacidosis in individuals with type 1 diabetes (T1D) ultimately changed T1D from a terminal disease into a manageable chronic illness [1,2]. One hundred years later, there have been significant improvements in T1D outcomes, but there is still no cure for T1D. These improvements were directly facilitated by advancements in insulin delivery (figure 1). Results from the Diabetes Control and Complications trial (DCCT) were reported in 1993 and illustrated that intensive insulin therapy, using three or more insulin injections or insulin pump therapy, was effective at reducing long-term complications of diabetes [3]. As a result, life expectancy for individuals with T1D has improved substantially, although is still on average 8 years less than someone without T1D [4,5].

The DCCT also demonstrated that achieving tighter glycemic targets in adolescents and adults is limited by insulin-induced hypoglycemia [6–8]. Younger children are likely at an even higher risk of hypoglycemia [9]. Additionally, hypoglycemia and fear of hypoglycemia are barriers to exercise, which itself has glycemic and cardiovascular benefits and reduces mortality [10–15]. In the thirty years following the initial DCCT reports, iatrogenic hypoglycemia is still the main limiting factor in achieving normoglycemia in individuals with T1D [16].

Insulin is safely and effectively administered for therapeutic use via intravenous, subcutaneous and inhaled routes [17,18]. Intravenous insulin is reserved for use in treatment of diabetic ketoacidosis, during major surgery, and in critical illness [19–21]. Intraperitoneal insulin using implantable pumps or percutaneous port systems is indicated when subcutaneous or inhaled routes are not possible or effective, although this is not widely available outside of Europe [22,23]. Intramuscular injections should be avoided due to their propensity to precipitate severe hypoglycemia [24,25]. Oral insulin is currently therapeutically ineffective due to enzymatic degradation by the gut [26]. It continues to be explored for a potential role in delaying or preventing T1D by inducing immune tolerance [27–30].

## 2. Updates to injectable insulin

The first insulin injections were subcutaneous injections of animal-derived insulin and given to prevent severe metabolic decompensation associated with insulinopenia [1]. The goal of insulin therapy has progressed from preventing coma and death to achieving near-normal glucose levels. This has required modifications to insulin’s structure and concentration, which were undertaken almost immediately after therapeutic use of insulin had begun [31]. Modifications to prolong the duration of insulin action were made as early as the 1930s and continue today [32,33]. Purification processes continued to improve; semi-synthetic insulin, which was less immunogenic than pure animal insulin, was developed in the early 1980s [34]. Recombinant DNA (rDNA) human insulin became a reality shortly after, which resulted in a purer, less immunogenic product and enabled large scale production [35]. Modifications to create rapid acting analog formulations became available in the 1990s and continue to be improved upon today [36].

Basal-bolus therapy is standard-of-care in T1D and can be achieved using regular human insulin or rapid-acting insulin analogs (lispro, aspart, glulisine) for prandial coverage [37,38]. Rapid-acting analogs are generally preferred for prandial insulin in multiple daily injection (MDI) regimens as they offer a reduction in hypoglycemia alongside decreased post-prandial blood glucose (BG) [39–43]. They have an onset of action in 15 to 20 minutes and peak action in 1-2 hours [44–48]. Their pharmacokinetics allow for increased dosing flexibility over older regimens. Additionally, they can be used in traditional insulin pump therapy and hybrid-closed loop (HCL) therapy, described in further detail in sections 3 and 5. The standard U-100 concentrations are available in vials, cartridges, and pre-filled insulin pens [44–46]. A concentrated form of rapid acting insulin (lispro U-200) exists and can be prescribed as pre-filled pens only [49]. The main benefit of concentrated lispro, which is bioequivalent to U-100 lispro, is the ability to deliver lower volumes of insulin [50]. Lower injected volumes may decrease injection site pain [51].

Basal insulin therapy has undergone a similarly impressive transformation. Neutral protamine Hagedorn (NPH) insulin, lente and ultralente as basal insulin therapy have been improved upon with peakless long and ultra-long-acting basal insulins (detemir, glargine, degludec, discussed in the following paragraph). Adult data demonstrates better glucose control with less hypoglycemia on basal-bolus regimens using the previously discussed rapid-acting analogs with newer long-acting insulin analogs compared to NPH insulin/regular human insulin [52,53]. Injection regimens with NPH and/or regular human insulin are still used in T1D and have the benefit of decreasing the injection burden and can be employed in situations where children do not have adequate supervision to inject insulin midday [38,54].

Glargine is an rDNA insulin with modifications made to the amino acid structure that shift the isoelectric point, causing it to precipitate in tissue after injection and extending its duration of action to 24 hours [55]. It comes in both U-100 and U-300 concentrations, with the U-300 concentration demonstrating even longer duration of action and a more even glucodynamic profile compared to U-100 [56]. As such, U-100 and U-300 glargine are not bioequivalent and a direct unit-per-unit conversion may not apply [57]. Detemir is also a modified rDNA insulin with an additional chemical modification (acylation) that allows it to bind to albumin, prolonging its absorption [58]. Smaller doses of detemir appear to last about 20 hours, with higher doses (0.6 to 1.6U/kg) lasting about 24 hours in adults with T2D [59]. Insulin degludec (Tresiba, NovoNordisk, Bagsvaerd, Denmark) is a new, ultra-long-acting basal insulin with a glucose lowering effect lasting 42 hours after injection [60]. It is also an rDNA insulin with both amino acid substitutions and a chemical modification (acylation via glutamic acid linker) [61]. When compared to insulin glargine, degludec has comparable efficacy but demonstrates less nocturnal hypoglycemia in adults [62,63]. Nocturnal hypoglycemia and hyperglycemia with ketosis are significantly reduced in pediatric patients when comparing degludec to detemir [64]. Degludec is available in U-100 and U-200 concentrations, which are bioequivalent [65].

### Updates to subcutaneous insulin- even faster onset

Rapid-acting insulin analogs, as discussed above, provide significant improvement in insulin delivery, however they are still too slow in onset to match the physiologic insulin secretion pattern in individuals without T1D. They must be taken well in advance of eating to have maximal impact on post-prandial hyperglycemia, which is difficult especially for young children [66]. Faster-acting aspart (Fiasp, NovoNordisk, Bagsvaerd, Denmark), a modified version of aspart, has more rapid onset in the circulation with greater glucose lowering effects during the first 2 hours than traditional aspart [66]. It was approved by the FDA in 2017 for adult use and subsequently was approved for adult use in insulin pumps in 2019 [67]. It has been demonstrated safe and effective in children with T1D [68] and was expanded to pediatric use in 2020 [69]. Ultra-rapid lispro (Lyumjev, Eli Lilly, Indiana, US), a modified version of lispro, has a similarly shifted pharmacokinetic and glucodynamic profile [70]. Ultra-rapid lispro was FDA approved for adults in June 2020 [71]. A phase III trial is expected to conclude in mid 2021 for pediatrics (NCT03740919). Compatibility with insulin pumps has been demonstrated [72], but it is not approved for use in insulin pumps at the time of submitting this paper.

### Insulin Pens

Insulin pens were first introduced in 1987 and partially address quality of life and diabetes outcome-related barriers to insulin delivery with vial and syringe (including convenience, dosage, pain, and hypoglycemia) [73]. Insulin pens contain pre-filled insulin, and a pen needle is attached for each injection. Accurate doses of insulin can be easily measured using a dial. The first digital insulin pen, the HumaPen Memoir, debuted in 2007 and allowed recall of the date, time, and recent insulin dosages [74]. The first smart insulin pen (InPen; Companion Medical Inc., San Diego, CA) launched in the US ten years later and syncs to a smartphone application. The application can be programmed with insulin-to-carbohydrate (I:C) ratios, insulin sensitivity factors (ISF), BG targets, and duration of insulin action (DIA) which culminate in dosage suggestions similar to pump bolus calculators. Residual bolus insulin is tracked and accounted for to mitigate insulin stacking. Fixed dosing and meal-estimation can be used in place of I:C ratios in the dose calculator settings. Settings can be varied by time of day [75].

## 3. Traditional “Open loop” Insulin pumps

MDI remains the predominant mode of insulin delivery worldwide, although continuous subcutaneous infusion (CSII) via insulin pump continues to increase and varies widely by age and population [76–78]. Insulin pumps fall into two broad categories of medical devices: conventional pumps and patch pumps. Conventional pumps have a housing (which contains the insulin, electronics, pump and battery), a subcutaneous catheter, and tubing which connects the insulin pump to the catheter [79]. Patch pumps are worn on the skin and house the insulin and pump mechanisms in a small contained device [79,80]. Both types of traditional pumps are based on an open-loop insulin delivery system, with no automation of insulin delivery based on BG levels. As such, traditional pumps can be used with or without continuous glucose monitoring (CGM) [80]. An excellent historical review of insulin pumps is provided by Alsaleh et al [80].

Current traditional insulin pumps allow for settings to be programmed by the provider/user and have built-in bolus calculators, which have demonstrated improvements in glycemic control and patient satisfaction [81,82]. Programmable settings are similar to the InPen discussed in Section 2 and include insulin-to-carbohydrate (I:C) ratio(s), basal rate(s), duration of insulin action/insulin action time(s) (DIA), insulin sensitivity factor(s) (ISF), and correction target(s) [83–86]. These settings can be varied throughout the day to account for changes in insulin needs and insulin sensitivity. In traditional pump therapy, DIA is used to calculate the remaining insulin on board at any given time after a bolus [83–85]. These settings can improve safety in insulin delivery and can help mitigate insulin stacking, which could lead to hypoglycemia. These settings are used to some degree in the various automated insulin delivery systems, which are discussed in sections 5 and 7.

Typical insulin pumps use only rapid-acting or faster-acting rapid-acting insulin. CSII may modestly reduce HbA1c without sufficient evidence to infer improvements in glycemic outcomes such as hypoglycemia when compared to MDI [87]. Several studies cite quality of life improvements on CSII therapy [88–90]. Additional benefits to CSII include the ability to use extended, dual, or square-wave boluses to more closely mimic absorption of mixed macronutrient content meals [91]. While diabetic ketoacidosis (DKA) is of theoretical concern on CSII given the sole use of rapid-acting insulin, recent studies do not demonstrate significant differences in rates of DKA between CSII and MDI. This risk estimate is limited due to risk of bias in most clinical studies [92,93]. Real-world data also do not favor increases in DKA in CSII users but may reflect CSII prescribing practices [78].

## 4. Partial automation part 1 – hypoglycemia mitigation. The evolution of low glucose suspend (LGS) and predictive low glucose suspend (PLGS)

Iatrogenic hypoglycemia continues to limit achievement of normoglycemia in T1D [16]. Additionally, fear of hypoglycemia is common in individuals with T1D and parents of children with T1D with significant impact on quality of life [94] and conflicting evidence on glycemic control [95,96]. Integration of CGM with CSII allowed for low glucose alarms and subsequently the birth of sensor-augmented pump therapy (SAP) systems with low-glucose suspend (LGS). LGS systems suspend insulin delivery when the CGM-reported BG hits a pre-set hypoglycemia threshold. Basal insulin typically remains suspended for a maximum fixed time interval if no action is taken [97]. Studies of LGS demonstrated stable glycemic control with improvements in hypoglycemia [98,99].

Introduction of algorithms used to forecast future hypoglycemia based on CGM trends and insulin-on-board led to predictive low glucose suspend (PLGS), including the MiniMed 640G (Medtronic, Northridge, California) and the Basal-IQ system (Tandem Inc., San Diego, California) [100,101]. Basal insulin delivery is shut off when the predicted or actual CGM value reaches a pre-specified hypoglycemia threshold and may remain suspended for a fixed time interval (Basal-IQ, 640G), may stay suspended until the CGM glucose value starts to rise (Basal-IQ), or a future predicted BG is above a pre-specified threshold (Basal-IQ, 640G) depending on the system [100,101]. PLGS demonstrates benefits over LGS, including less time spent < 70 mg/dL. PLGS also demonstrates reduction in fear of hypoglycemia and improvements in sleep quality while maintaining stable glycemic control [102].

## 5a. Commercially-available Hybrid closed loop systems (HCL)

Hybrid closed loop systems (HCL, also called artificial pancreas) are insulin delivery systems that automate insulin delivery in addition to augmenting or suspending insulin for actual or predicted hypoglycemia. They employ algorithms that integrate readings from CGM along with insulin-on-board (with other nuances, out of the scope of this article). The outcome is dynamic modulation of insulin administration to keep BG at a prespecified target or in a prespecified target range. HCL are not completely closed systems, as carbohydrates must still be counted and manually entered by the user. Each system uses a different algorithm running on a different device (i.e., pump or cellphone) and employs different CGM(s). Each system thus has different adjustable settings and reasons for auto mode exits [103]. Commercially-available, soon-to-be-available, and do-it-yourself systems are summarized in Table 1. More details about do-it-yourself systems can be found in section 5b.

The first FDA-approved HCL system available was Minimed 670G (Medtronic, Northridge, California). When in auto mode, automated basal rates are modulated to achieve the preset target. The user/provider can adjust I:C ratios and DIA when in auto mode. The auto mode target is set at 120 mg/dL unless a temporary (exercise) target is set, which raises the target to 150 mg/dL. ISF is autosensed using historical data, so extraneous insulin entries and/or significant variability in insulin sensitivity may affect performance. The pump may recommend a user-initiated bolus if the BG is greater than or equal to 150 mg/dL. Users cannot employ square wave, dual wave, or other types of advanced boluses when in auto mode [84]. The 770G was approved last year with an expanded age indication and allowed for remote data visualization [104]. The 780G is not yet FDA approved but adds in autocorrective boluses with an updated algorithm. It is expected to work with an improved CGM that will only require calibration on the first day of use. The 780G features a default target of 100 mg/dL, with an option to increase to 120 mg/dL. All three MiniMed systems have demonstrated safety and efficacy [105–107]. Further improvements to hyperglycemia and TIR without increased hypoglycemia are noted when comparing the 780G to the 670G [105,108]. Real world data from the 670G demonstrates that percentage of time spent in auto mode declined significantly over the first year with a main barrier to usage being CGM-related issues [109,110].

The Tandem Control-IQ system (Tandem Inc., San Diego, California) has the ability to give automated correction boluses [111]. However, one still must bolus for meals. In normal Control-IQ mode, user/provider-set basal rates are adjusted to keep BGs in the target range of 112.5-160 mg/dL. Insulin delivery is suspended if BG is predicted to go below 70 mg/dL 30 minutes into the future or does go below 70 mg/dL. Automatic corrections are given if BG is predicted to be above 180 mg/dL in the next 30 minutes and are given up to once per hour using a correction target of 110 mg/dL. Sixty percent of the predicted insulin correction is administered with automatic correction boluses. The ISF can be adjusted by the user/provider— ISF, IOB, and TDD drive the algorithm’s maximum insulin delivery rate calculation. Extended boluses can be set for 2 hours duration in Control-IQ mode. The user can enable activity modes (i.e., sleep or exercise) which modify the BG target range. No automatic boluses are given during sleep mode [83]. Pivotal trials using Control-IQ demonstrated safety and efficacy as compared to SAP in children and adults, with significant improvements in TIR and HbA1c [111,112]. Adults also saw decreased hypoglycemia [111]. Median percent time in automation remained over 90% at one year of real world use with this system [113].

Omnipod (Insulet Corporation, Boston, Massachusetts) has completed their pivotal trial for the Omnipod 5 automated glucose control system (NCT04196140) in individuals aged 6-70. Data are pending FDA review, but currently available data demonstrate improvements in HbA1c and TIR in pediatric and adult participants, with adults also demonstrating a decreased time spent in hypoglycemia [114]. Free-living trials have also demonstrated safety and efficacy of this system [115]. Benefits of this system include tubeless automated insulin delivery and adjustable BG targets that can be varied by time of day [116].

The CamAPS system is the only HCL system currently approved for use in pregnancy, receiving a CE mark for use in 2020 [117]. A thorough review of commercially available hybrid closed-loop systems in the UK provides additional details on the CamAPS FX system (CamDiab Ltd, Cambridge, UK) [118–120] . The DBLG1 system (Diabeloop, Grenoble, France) recently completed a real-world efficacy trial with promising results [121]. Additional details about the system can be found in the pilot study (Benhamou et al) [122].

There are other HCL algorithms in development, both in academic and commercial settings. It is beyond the scope of this article to review them all in detail. Some of these include dual hormone (i.e., insulin and glucagon) algorithms, which are briefly reviewed in section 7. Beta Bionic’s iLet (Beta Bionics, Boston, Massachusetts, USA), which has single and dual hormone options has an ongoing phase 3 clinical trial (NCT04200313) for the insulin-only version of their pump, and enrolled adults and children age 6+. The SAFE-AP system is a single-hormone HCL controller that includes carbohydrate recommendations as an additional control input. This can be used with both announced and unannounced exercise and has been demonstrated to maintain BGs within target range after both unannounced and announced heavy physical activity. This algorithm is programmed to ensure that the counter-regulatory effect of the rescue carbohydrates does not trigger additional insulin release [123].

## 5b. Do-it-yourself (DIY) Hybrid closed loop systems

Frustrated with the pace of discovery, unaffordability, and lack of customization, the #WeAreNotWaiting movement developed do-it-yourself artificial pancreas systems (DIYAPS) [124]. The three main DIYAPS systems include OpenAPS (algorithm runs on a small computer), AndroidAPS (algorithm runs on an android phone, same base algorithm as OpenAPS), and Loop (algorithm runs on an iPhone and uses a hardware radio bridge communication device). The FreeAPS branch of Loop, which offered some unique features, has been frozen as the developers work on a new DIYAPS called FreeAPS X [125]. Similar to the commercially-available systems, the algorithms collect and analyze data related to glucose, insulin, and carbohydrates to predict future glucose levels and automate insulin delivery in response. There are additional customizations and forked branches. A comprehensive review of these systems is provided by Kesavadev et al [126]. At the of the time of article submission, over 2200 people worldwide were using DIYAPS [127].

The Loop Observational Study (NCT03838900) recently completed after enrolling 1212 participants using Loop. They collected device data via Tidepool (Tidepool Project, Palo Alto, California), HbA1c, self-reported adverse events, self-reported device issues, and psychosocial/user experience data. Interval data were presented at the Advanced Technologies and Treatments for Diabetes meeting in 2020 on 873 Loop users. The baseline mean TIR for users was fairly high at 67% but improved significantly to 73% with corresponding reductions in HbA1c. They reported low baseline and follow up hypoglycemia rates with additional improvements in diabetes distress, fear of hypoglycemia, and sleep quality [128]. Tidepool has adapted the Loop app for commercial use as an interoperable glycemic controller, meaning their application will work across platforms with multiple insulin pumps and CGMs [129]. The 510(k) was submitted to FDA in December 2020 using data from the aforementioned Loop Observational Study[128]. In silico trials of the OpenAPS algorithm using University of Virginia’s Padova T1D simulator demonstrated safety and efficacy, with optimal performance using the automated bolus version (oref1 algorithm)[130]. There are few randomized control trials evaluating DIYAPS, but an OpenAPS repository includes promising real-world data [127]. A randomized clinical trial of AndroidAPS (the CREATE trial), comparing this to SAP therapy is underway. An excellent review on outcomes of DIYAPS studies is provided by Jennings et all [131].

## 6. Technosphere inhaled insulin

Inhaled human insulin (Afrezza, Valencia, California) was approved in 2014 for adults with diabetes as an alternative prandial insulin. It follows Exubera (Pfizer, New York, NY, USA), which is an inhaled insulin approved in 2006. Production of Exubera was voluntarily discontinued shortly thereafter due to low sales [132]. Safety and pharmacokinetic studies of Afrezza in pediatric patients were recently completed in children aged 8-17 in two age cohorts (NCT02527625) but it is not yet approved for pediatric use. Afrezza comes in cartridges that can be dosed in 4-unit increments [133]. Multiple cartridges are required for doses exceeding 12U. Spirometry is required prior to initiation of therapy as it is contraindicated in patients with chronic lung disease. Afrezza has an onset of action of less than 15 minutes with a mean peak action of 50 minutes in adults [133]. The duration of action varies by dose, ranging from 90 minutes with smaller doses to 270 minutes at larger doses [133]. Individuals report improved satisfaction and quality of life using inhaled insulin as compared to injectable insulin [134,135]. Inhaled insulin has been associated with lower weight gain in type 2 diabetes and less hypoglycemia compared to subcutaneous insulin but generally demonstrates lower glycemic efficacy compared to subcutaneous insulin therapy [136]. Studies using CGM to monitor glycemia in T1D using inhaled insulin are sparse as are long-term trials evaluating glycemia [18]. The most common pulmonary symptom associated with use of inhaled insulin is non-productive cough [136]. A recent study comparing pre-prandial doses of inhaled insulin versus subcutaneous CSII insulin boluses in conjunction with HCL therapy have demonstrated improvements in early glycemic excursions with utilization of inhaled insulin, consistent with its glucodynamic action. Additional research is needed to determine efficacy, safety, and patient satisfaction of this inhaled/HCL combination insulin dose strategy [137].

## 7. The future of insulin administration– insulin adjuncts, additional improvements to insulin, complete closed loop, dual-hormone pumps

### Sodium-glucose cotransporter (SGLT) inhibitors

SGLT-2 is present in the proximal tubule of the kidney, and SGLT-1 is present in both the kidneys and the intestine. SGLT inhibitors are a class of oral medication that eliminate glucose reabsorption in either or both of these transporters, which increases urinary glucose excretion, decreases intestinal glucose absorption, and as such decrease BG in an insulin-independent manner [138]. Results of the EASE, DEPICT, and inTandem trials demonstrated that selective SLGT2 inhibitors are effective adjuncts for glucose lowering therapy in adults with T1D [139–141]. These trials also noted improved weight loss without hypoglycemia. The major concern in the use of SGLT inhibitors is euglycemic DKA, postulated to occur via a starvation mechanism [142]. Meta-analysis of SGLT inhibitors in T1D have similarly demonstrated efficacy, with primary adverse events being DKA and genital infections [143]. Continuous ketone monitoring would allow for safer utilization of SGLT agonists, facilitate the safe use of very-low carb diets, as well as provide adjunctive data to closed-loop algorithms. Microneedle technology employing a NAD-dependent dehydrogenase-based electrochemical biosensor has demonstrated the ability to detect real-time levels of ß-hydroxylbutyrate along with glucose and lactate [144].

### Insulin updates

The most common cause of DKA is insulin non-adherence, and current insulin regimens are cumbersome [145]. Simplified treatment regimens have shown improvements in patient-reported outcomes in type 2 diabetes, but direct studies in T1D are difficult due to the necessity of insulin therapy [146]. Once-weekly insulin treatment with insulin icodec was demonstrated to be effective at lowering HbA1c levels in adults with type 2 diabetes [147]. Phase 3 trials of insulin icodec compared to degludec are being studied in adults with type 1 diabetes (NCT04848480). Weekly insulin for T1D may offer improvements in quality of life and decrease DKA due to insulin omission. The biggest weakness to weekly-dosed insulin is the inflexibility of dosing, which has major implications in active and growing individuals. This may make icodec of limited benefit for the pediatric population, who is likely to benefit most from simplifying insulin delivery.

### Hybrid closed-loop and fully closed loop insulin pump therapy

Current rapid acting insulin formulations do not have fast enough onset to allow for completely successful total automation. Simple meal announcement without carbohydrate counting using insulin-only strategies may alleviate some of the mental burden of T1D and improve glycemic outcomes and quality of life. Use of faster-acting insulin analogs discussed above in HCL is underway [148–151]. Faster acting insulins could potentially allow for simplification of meal bolusing or elimination of meal announcement all together [152]. DIY options (i.e. OpenAPS’s advanced dosing features) already exist for unannounced meals [153]. Dual-hormone systems, discussed immediately below, may also make full automation possible.

### Dual-hormone closed-loop therapy (insulin/glucagon)

The advent of liquid stable glucagon has enabled dual hormone (DH) closed-loop systems to become achievable [154–156]. Several research groups are working on automated DH algorithms and devices, which administer both insulin and glucagon [157–161]. Early feasibility studies show promise for both improved glycemic control as well as hypoglycemia mitigation over PLGS and HCL systems including in situations with a propensity to trigger hypoglycemia, such as exercise [162,163]. In addition to hypoglycemia, DH algorithms show promise in eliminating precise carbohydrate counting although do seem to operate best when using simple meal announcements with estimated meal size [162]. Adverse outcomes of using micro-doses of glucagon primarily include nausea, vomiting, and headache [164].

### Other dual-hormone options (pramlintide/insulin, GLP-1 agonists/insulin)

Pramlintide, an analogue of amylin, can be co-injected with insulin to delay gastric emptying and suppress glucagon secretion and has been demonstrated to improve post-prandial hyperglycemia in T1D. Pramlintide co-administration with rapid-acting insulin in HCL systems has been demonstrated to mitigate meal-related glycemic excursions [165]. Co-administration of faster-acting insulin aspart and pramlintide with only simple meal announcement demonstrated feasibility, and the results of the non-inferiority trial to HCL with standard carbohydrate counting completed in February 2020 (NCT02814123) [152]. Liraglutide, a GLP-1 agonist, may reduce post-prandial glucose excursions and HbA1c in T1D but also may increase hypoglycemia and ketosis [166,167], making it unlikely to gain widespread use in T1D management.

### Improvements for exercise

Management of T1D and exercise remains challenging due to increases in insulin sensitivity and increased insulin-independent glucose uptake into muscles which can persist long after the exercise has completed [168–170]. DH-closed loop systems discussed above show promise in mitigating early and late-onset exercise-induced hypoglycemia in T1D [157,171,172]. Incorporation of heart-rate detection, and lactate and ketone levels into closed-loop algorithms may also help overcome challenges associated with exercise and T1D [144,173–176].

## 8. Caveats

Despite significant advancements in insulin and related diabetes technology, a minority of patients with T1D are meeting the glycemic targets known to reduce complications [78]. Further work needs to be done to explore barriers to achievement of these goals, especially in the adolescent population who have seen worsening glycemic control despite increasing technology uptake [78].

Additionally, equity in diabetes care and care delivery needs to be addressed, as related variables are often absent from trials [92]. Despite an overall rise in insulin pump use [which is associated with improvements in quality of life], non-Hispanic Black youth, Hispanic youth, and American Indian/Alaskan Native youth are significantly less likely to be on insulin pump therapy independent of socioeconomic status [177]. CGM in conjunction with CSII, especially when accounting for PLGS and HCL functionality have demonstrated superiority in many studies and highlights the need to understand and address this inequity [178].

Addressing socioeconomic barriers will also be crucial as the management of T1D continues to evolve. The cost of insulin can be prohibitive [179]. Rapid acting analogs are cost-effective in the treatment of T1D however may be unattainable for uninsured patients [180,181]. Heterogeneity exists in reimbursement for diabetes technology [182]. As such, patients and their families may have difficulties accessing more advanced diabetes features. Pre-existing gaps in diabetes care and outcomes are likely to widen [183,184]. Advanced diabetes devices are difficult to learn, maintain and manage, and different educational frameworks will be needed to allow for a wide spectrum of users to achieve success. Mentorship programs and telehealth show promise in addressing health disparities in T1D [185,186]. Further research in optimal education and care delivery are necessary as diabetes technology becomes more advanced.

## 9. Conclusions

Insulin administration has evolved dramatically over the last 100 years. However, with no imminent cure for T1D, further optimization of insulin delivery is necessary. Iatrogenic hypoglycemia is still therapeutically problematic and impairs quality of life. Automated insulin delivery shows great promise in helping patients achieve glycemic targets while mitigating hypoglycemia, but is not perfect nor universally accessible. Research is necessary to identify and rectify barriers to uptake and continued utilization of more advanced methods of insulin administration. More robust head-to-head comparisons of automated insulin delivery methods using representative populations in real-world conditions as well as pragmatic trials will facilitate further improvements and allow for a more patient-centered approach to T1D.

## Figures and Tables

**Table 1.** Comparison of Insulin Pump systems

Single Hormone HCL System	Compatible CGM(s)	BG target in standard automated modes	BG targets in activity modes	Method(s) of insulin adjustment	User-adjustable settings in automated mode	Settings that cannot be adjusted in automated mode	FDA-approved?	Insulin requirements for auto mode
MiniMed 670G	Guardian 3, calibration required	120 mg/dL	Exercise: 150 mg/dL	Basal modulation; pump may recommend bolus if BG ≥ 150 mg/dL but user must accept. System calculates the TDD over the past 6 and uses this to inform the basal rates and ISF.	I:C ratio DIA	Basal rates Target (except for exercise) ISF	Yes, age 7+	8U-250U TDD
MiniMed 770G	Guardian 3, calibration required	120 mg/dL	Exercise: 150 mg/dL	Basal modulation; pump may recommend bolus if BG ≥ 150 mg/dL but user must accept. System calculates the TDD over the past 6 days and uses this to inform the basal rates and ISF.	I:C ratio DIA	Basal rates Target (except for exercise) ISF	Yes, age 2+	8U-250U TDD
MiniMed 780G	Guardian 3, planned release of updated sensor requiring no calibration	100 mg/dL, can increase to 120 mg/dL	Exercise: 150 mg/dL	Basal modulation and automatic correction boluses q5 minutes. System calculates the TDD over the past 6 days at midnight and uses this to inform the basal rates and ISF.	I:C ratio DIA Target	Basal rates ISF	Not yet, (approved in Europe in June 2020); likely to start out at 7+	8U-250U TDD
Tandem Control-IQ	Dexcom G6	112.5 mg/dL – 160 mg/dL; Correction target is 110 mg/dL	Sleep: 112.5 mg/dL – 120 mg/dL Exercise: 140 mg/dL – 160 mg/dL	Basal modulation of pre-set basal rates and automatic correction boluses q1 hour of 60% predicted need (correction boluses not administered in sleep mode).	I:C ratio ISF Basal rate TDD (this constrains the max basal rates) Target	DIA Correction target, can tighten range by running sleep	Yes, age 6+	10U TDD minimum
CamAPS FX	Dexcom G6	Default is 104 mg/dL but is adjustable (80-200 mg/dL); can be varied by time of day	Boost: for periods of increased insulin needs (i.e. atypical food intake, stress, illness) Ease off: for when less insulin is needed (i.e. exercise); can be set in the future	Basal modulation	I:C ratio and ISF are used by the bolus calculator for meals and for user-initiated correction boluses in auto mode	Basal rates	No, but approved in UK/EU for age 1+	5U-350U TDD; at least 10kg
Omnipod 5	Dexcom G6	110-150 mg/dL in 10 mg/dL increments; can be varied by time of day	Hypoprotect: 150 mg/dL	Basal modulation. Autobasals are based on estimated and then actual TDD after initialization.	Target ISF,DIA (used for user-initiated correction boluses in auto mode, not autodelivered insulin)	Basal rates	Submitted	None mentioned
Tidepool Loop	Many	Likely to be adjustable	Likely to be adjustable	Basal modulation	I:C ratio ISF Basal rate Target	DIA	No	None studied, seeking FDA approval from DIY Loop studies
Loop	Many	Adjustable	Adjustable	Basal modulation of the pre-set basal rates. Autobolus branch gives automatic correction boluses.	I:C ratio ISF, Basal rate Target	DIA (you choose the model based on age and type of insulin you use, can be modified with code adjustments)	No - DIY	None studied
AndroidAPS	Many	Adjustable	Adjustable	Basal modulation of the pre-set basal rates. Oref1 can give automated boluses.	I:C ratio ISF, Basal rate DIA, Target Advanced settings (max iob, autosensing, insulin curves, etc.)	N/A	No - DIY	None studied
OpenAPS	Many	Adjustable	Adjustable	Basal modulation of the pre-set basal rates. Oref1 can give automated boluses.	I:C ratio ISF, Basal rate Basal rate DIA, Target Advanced settings (max iob, autosensing, insulin curves, etc.)	N/A	No - DIY	None studied

Summary of currently and soon-to-be available single hormone hybrid closed loop insulin delivery systems. HCL, hybrid closed loop; BG, blood glucose; CGM, continuous glucose monitor; I:C, insulin-to-carbohydrate ratio; ISF, insulin sensitivity factor; DIA, duration of insulin action; iob, insulin on board; DIY, do-it-yourself; TDD, total daily dose.

## References

[1] Banting FG; Best CH; Collip JB; Campbell WR; Fletcher AA Pancreatic Extracts in the Treatment of Diabetes Mellitus. Canadian Medical Association journal 1922, 12, 141–146. 20314060 PMC1524425

[2] Banting FG; Campbell WR; Fletcher AA Further Clinical Experience with Insulin (Pancreatic Extracts) in the Treatment of Diabetes Mellitus. Br Med J 1923, 1, 8–12, doi:10.1136/bmj.1.3236.8. 20770964 PMC2316395

[3] Diabetes, C.; Complications Trial Research, G.; Nathan DM; Genuth S; Lachin J; Cleary P; Crofford O; Davis M; Rand L; Siebert C The effect of intensive treatment of diabetes on the development and progression of long-term complications in insulin-dependent diabetes mellitus. N Engl J Med 1993, 329, 977–986, doi:10.1056/NEJM199309303291401. 8366922

[4] Miller RG; Secrest AM; Sharma RK; Songer TJ; Orchard TJ Improvements in the life expectancy of type 1 diabetes: the Pittsburgh Epidemiology of Diabetes Complications study cohort. Diabetes 2012, 61, 2987–2992, doi:10.2337/db11-1625. 22851572 PMC3478551

[5] Heald AH; Stedman M; Davies M; Livingston M; Alshames R; Lunt M; Rayman G; Gadsby R Estimating life years lost to diabetes: outcomes from analysis of National Diabetes Audit and Office of National Statistics data. Cardiovasc Endocrinol Metab 2020, 9, 183–185, doi:10.1097/xce.0000000000000210. 33225235 PMC7673790

[6] Epidemiology of severe hypoglycemia in the diabetes control and complications trial. The DCCT Research Group. The American journal of medicine 1991, 90, 450–459. 2012085

[7] Effect of intensive diabetes treatment on the development and progression of long-term complications in adolescents with insulin-dependent diabetes mellitus: Diabetes Control and Complications Trial. Diabetes Control and Complications Trial Research Group. The Journal of pediatrics 1994, 125, 177–188, doi:10.1016/s0022-3476(94)70190-3. 8040759

[8] Hypoglycemia in the Diabetes Control and Complications Trial. The Diabetes Control and Complications Trial Research Group. Diabetes 1997, 46, 271–286. 9000705

[9] Cengiz E; Xing D; Wong JC; Wolfsdorf JI; Haymond MW; Rewers A; Shanmugham S; Tamborlane WV; Willi SM; Seiple DL, Severe hypoglycemia and diabetic ketoacidosis among youth with type 1 diabetes in the T1D Exchange clinic registry. Pediatric diabetes 2013, 14, 447–454, doi:10.1111/pedi.12030. 23469984 PMC4100244

[10] Brazeau AS; Rabasa-Lhoret R; Strychar I; Mircescu H Barriers to physical activity among patients with type 1 diabetes. Diabetes care 2008, 31, 2108–2109, doi:10.2337/dc08-0720. 18689694 PMC2571055

[11] Roberts AJ; Taplin CE; Isom S; Divers J; Saydah S; Jensen ET; Mayer-Davis EJ; Reid LA; Liese AD; Dolan LM, Association between fear of hypoglycemia and physical activity in youth with type 1 diabetes: The SEARCH for diabetes in youth study. Pediatric diabetes 2020, 10.1111/pedi.13092, doi:10.1111/pedi.13092. PMC7855399 32738012

[12] Jabbour G; Henderson M; Mathieu ME Barriers to Active Lifestyles in Children with Type 1 Diabetes. Can J Diabetes 2016, 40, 170–172, doi:10.1016/j.jcjd.2015.12.001. 27038139

[13] Herbst A; Kordonouri O; Schwab KO; Schmidt F; Holl RW Impact of physical activity on cardiovascular risk factors in children with type 1 diabetes: a multicenter study of 23,251 patients. Diabetes care 2007, 30, 2098–2100, doi:10.2337/dc06-2636. 17468347

[14] Miller RG; Mahajan HD; Costacou T; Sekikawa A; Anderson SJ; Orchard TJ A Contemporary Estimate of Total Mortality and Cardiovascular Disease Risk in Young Adults With Type 1 Diabetes: The Pittsburgh Epidemiology of Diabetes Complications Study. Diabetes care 2016, 39, 2296–2303, doi:10.2337/dc16-1162. 27654986 PMC5127232

[15] Kriska AM; LaPorte RE; Patrick SL; Kuller LH; Orchard TJ The association of physical activity and diabetic complications in individuals with insulin-dependent diabetes mellitus: the Epidemiology of Diabetes Complications Study--VII. J Clin Epidemiol 1991, 44, 1207–1214, doi:10.1016/0895-4356(91)90153-z. 1941015

[16] Cryer PE Banting Lecture. Hypoglycemia: the limiting factor in the management of IDDM. Diabetes 1994, 43, 1378–1389, doi:10.2337/diab.43.11.1378. 7926315

[17] Frid AH; Kreugel G; Grassi G; Halimi S; Hicks D; Hirsch LJ; Smith MJ; Wellhoener R; Bode BW; Hirsch IB, New Insulin Delivery Recommendations. Mayo Clinic proceedings 2016, 91, 1231–1255, doi:10.1016/j.mayocp.2016.06.010. 27594187

[18] Garg SK; Mathieu C; Rais N; Gao H; Tobian JA; Gates JR; Ferguson JA; Webb DM; Berclaz PY Two-year efficacy and safety of AIR inhaled insulin in patients with type 1 diabetes: An open-label randomized controlled trial. Diabetes technology & therapeutics 2009, 11 Suppl 2, S5–s16, doi:10.1089/dia.2009.0040. 19772449

[19] Wolfsdorf JI; Glaser N; Agus M; Fritsch M; Hanas R; Rewers A; Sperling MA; Codner E ISPAD Clinical Practice Consensus Guidelines 2018: Diabetic ketoacidosis and the hyperglycemic hyperosmolar state. Pediatric diabetes 2018, 19 Suppl 27, 155–177, doi:10.1111/pedi.12701. 29900641

[20] Jefferies C; Rhodes E; Rachmiel M; Chizo AJ; Kapellen T; Abdulla MA; Hofer SE ISPAD Clinical Practice Consensus Guidelines 2018: Management of children and adolescents with diabetes requiring surgery. Pediatric diabetes 2018, 19 Suppl 27, 227–236, doi:10.1111/pedi.12733. 30039617

[21] Pérez A; Ramos A; Carreras G Insulin Therapy in Hospitalized Patients. Am J Ther 2020, 27, e71–e78, doi:10.1097/mjt.0000000000001078. 31833876

[22] Rieger C; Kurz K; Mueller-Hoffmann W; Gehr B; Liebl A New Design of a Percutaneous Port System for Continuous Intraperitoneal Insulin Infusion. Journal of diabetes science and technology 2019, 13, 1158–1160, doi:10.1177/1932296819855425. 31195815 PMC6835186

[23] Spaan N; Teplova A; Stam G; Spaan J; Lucas C Systematic review: continuous intraperitoneal insulin infusion with implantable insulin pumps for diabetes mellitus. Acta diabetologica 2014, 51, 339–351, doi:10.1007/s00592-014-0557-3. 24595518

[24] Karges B; Boehm BO; Karges W Early hypoglycaemia after accidental intramuscular injection of insulin glargine. Diabetic medicine : a journal of the British Diabetic Association 2005, 22, 1444–1445, doi:10.1111/j.1464-5491.2005.01654.x. 16176210

[25] Frid A; Gunnarsson R; Güntner P; Linde B Effects of accidental intramuscular injection on insulin absorption in IDDM. Diabetes care 1988, 11, 41–45, doi:10.2337/diacare.11.1.41. 3276476

[26] Arbit E; Kidron M Oral insulin: the rationale for this approach and current developments. Journal of diabetes science and technology 2009, 3, 562–567, doi:10.1177/193229680900300322. 20144296 PMC2769870

[27] Krischer JP; Schatz DA; Bundy B; Skyler JS; Greenbaum CJ Effect of Oral Insulin on Prevention of Diabetes in Relatives of Patients With Type 1 Diabetes: A Randomized Clinical Trial. Jama 2017, 318, 1891–1902, doi:10.1001/jama.2017.17070. 29164254 PMC5798455

[28] Kumar V; Choudhry I; Namdev A; Mishra S; Soni S; Hurkat P; Jain A; Jain D Oral Insulin: Myth or Reality. Current diabetes reviews 2018, 14, 497–508, doi:10.2174/1573399813666170621122742. 28637407

[29] Sosenko JM; Skyler JS; Herold KC; Schatz DA; Haller MJ; Pugliese A; Cleves M; Geyer S; Rafkin LE; Matheson D, Slowed Metabolic Decline After 1 Year of Oral Insulin Treatment Among Individuals at High Risk for Type 1 Diabetes in the Diabetes Prevention Trial-Type 1 (DPT-1) and TrialNet Oral Insulin Prevention Trials. Diabetes 2020, 69, 1827–1832, doi:10.2337/db20-0166. 32439823 PMC7372067

[30] Assfalg R; Knoop J; Hoffman KL; Pfirrmann M; Zapardiel-Gonzalo JM; Hofelich A; Eugster A; Weigelt M; Matzke C; Reinhardt J, Oral insulin immunotherapy in children at risk for type 1 diabetes in a randomised controlled trial. Diabetologia 2021, 64, 1079–1092, doi:10.1007/s00125-020-05376-1. 33515070 PMC8012335

[31] Hirsch IB; Juneja R; Beals JM; Antalis CJ; Wright EE The Evolution of Insulin and How it Informs Therapy and Treatment Choices. Endocrine reviews 2020, 41, 733–755, doi:10.1210/endrev/bnaa015. 32396624 PMC7366348

[32] Quianzon CC; Cheikh I History of insulin. J Community Hosp Intern Med Perspect 2012, 2, doi:10.3402/jchimp.v2i2.18701. PMC3714061 23882369

[33] Gottlieb PA; Michels AW Advances in Diabetes Treatment - Once-Weekly Insulin. The New England journal of medicine 2020, 383, 2171–2172, doi:10.1056/NEJMe2031596. 33252874

[34] Markussen J; Damgaard U; Jørgensen KH; Sørensen E; Thim L Human monocomponent insulin. Chemistry and characteristics. Acta Med Scand Suppl 1983, 671, 99–105. 6349271

[35] Galloway JA Insulin treatment for the early 80s: facts and questions about old and new insulins and their usage. Diabetes care 1980, 3, 615–622, doi:10.2337/diacare.3.5.615. 7002515

[36] Tibaldi JM Evolution of insulin: from human to analog. The American journal of medicine 2014, 127, S25–38, doi:10.1016/j.amjmed.2014.07.005. 25282010

[37] 9. Pharmacologic Approaches to Glycemic Treatment: Standards of Medical Care in Diabetes-2020. Diabetes care 2020, 43, S98–s110, doi:10.2337/dc20-S009. 31862752

[38] Danne T; Phillip M; Buckingham BA; Jarosz-Chobot P; Saboo B; Urakami T; Battelino T; Hanas R; Codner E ISPAD Clinical Practice Consensus Guidelines 2018: Insulin treatment in children and adolescents with diabetes. Pediatric diabetes 2018, 19 Suppl 27, 115–135, doi:10.1111/pedi.12718. 29999222

[39] Holcombe JH; Zalani S; Arora VK; Mast CJ Comparison of insulin lispro with regular human insulin for the treatment of type 1 diabetes in adolescents. Clin Ther 2002, 24, 629–638, doi:10.1016/s0149-2918(02)85138-4. 12017407

[40] Deeb LC; Holcombe JH; Brunelle R; Zalani S; Brink S; Jenner M; Kitson H; Perlman K; Spencer M Insulin lispro lowers postprandial glucose in prepubertal children with diabetes. Pediatrics 2001, 108, 1175–1179, doi:10.1542/peds.108.5.1175. 11694699

[41] Nicolucci A; Ceriello A; Di Bartolo P; Corcos A; Orsini Federici M Rapid-Acting Insulin Analogues Versus Regular Human Insulin: A Meta-Analysis of Effects on Glycemic Control in Patients with Diabetes. Diabetes Ther 2020, 11, 573–584, doi:10.1007/s13300-019-00732-w. 31873857 PMC7048883

[42] Fullerton B; Siebenhofer A; Jeitler K; Horvath K; Semlitsch T; Berghold A; Plank J; Pieber TR; Gerlach FM Short-acting insulin analogues versus regular human insulin for adults with type 1 diabetes mellitus. Cochrane Database Syst Rev 2016, 2016, Cd012161, doi:10.1002/14651858.Cd012161. PMC6597145 27362975

[43] Melo KFS; Bahia LR; Pasinato B; Porfirio GJM; Martimbianco AL; Riera R; Calliari LEP; Minicucci WJ; Turatti LAA; Pedrosa HC, Short-acting insulin analogues versus regular human insulin on postprandial glucose and hypoglycemia in type 1 diabetes mellitus: a systematic review and meta-analysis. Diabetol Metab Syndr 2019, 11, 2, doi:10.1186/s13098-018-0397-3. 30622653 PMC6317184

[44] Lilly E US Prescribing Information — Humalog. Availabe online: https://pi.lilly.com/us/humalog-pen-pi.pdf (accessed on June 23).

[45] US Prescribing Information — Novolog. Availabe online: https://www.novo-pi.com/novolog.pdf (accessed on June 23).

[46] US Prescribing Information — Apidra. Availabe online: https://www.accessdata.fda.gov/drugsatfda_docs/label/2008/021629s015lbl.pdf (accessed on June 23).

[47] Lindholm A; Jacobsen LV Clinical pharmacokinetics and pharmacodynamics of insulin aspart. Clinical pharmacokinetics 2001, 40, 641–659, doi:10.2165/00003088-200140090-00002. 11605714

[48] Becker RH; Frick AD Clinical pharmacokinetics and pharmacodynamics of insulin glulisine. Clinical pharmacokinetics 2008, 47, 7–20, doi:10.2165/00003088-200847010-00002. 18076215

[49] Humalog 200 U/mL KwikPen. Availabe online: https://www.humalog.com/u200 (accessed on July 8).

[50] de la Peña A; Seger M; Soon D; Scott AJ; Reddy SR; Dobbins MA; Brown-Augsburger P; Linnebjerg H Bioequivalence and comparative pharmacodynamics of insulin lispro 200 U/mL relative to insulin lispro (Humalog^®^) 100 U/mL. Clin Pharmacol Drug Dev 2016, 5, 69–75, doi:10.1002/cpdd.221. 27119580 PMC5054907

[51] Heise T; Nosek L; Dellweg S; Zijlstra E; Præstmark KA; Kildegaard J; Nielsen G; Sparre T Impact of injection speed and volume on perceived pain during subcutaneous injections into the abdomen and thigh: a single-centre, randomized controlled trial. Diabetes, obesity & metabolism 2014, 16, 971–976, doi:10.1111/dom.12304. 24720741

[52] Hermansen K; Fontaine P; Kukolja KK; Peterkova V; Leth G; Gall MA Insulin analogues (insulin detemir and insulin aspart) versus traditional human insulins (NPH insulin and regular human insulin) in basal-bolus therapy for patients with type 1 diabetes. Diabetologia 2004, 47, 622–629, doi:10.1007/s00125-004-1365-z. 15298338

[53] Monami M; Marchionni N; Mannucci E Long-acting insulin analogues vs. NPH human insulin in type 1 diabetes. A meta-analysis. Diabetes, obesity & metabolism 2009, 11, 372–378, doi:10.1111/j.1463-1326.2008.00976.x. 19267715

[54] Chase HP; Dixon B; Pearson J; Fiallo-Scharer R; Walravens P; Klingensmith G; Rewers M; Garg SK Reduced hypoglycemic episodes and improved glycemic control in children with type 1 diabetes using insulin glargine and neutral protamine Hagedorn insulin. The Journal of pediatrics 2003, 143, 737–740, doi:10.1067/s0022-3476(03)00415-3. 14657818

[55] Heinemann L; Linkeschova R; Rave K; Hompesch B; Sedlak M; Heise T Time-action profile of the long-acting insulin analog insulin glargine (HOE901) in comparison with those of NPH insulin and placebo. Diabetes care 2000, 23, 644–649. 10834424 10.2337/diacare.23.5.644

[56] Becker RH; Dahmen R; Bergmann K; Lehmann A; Jax T; Heise T New insulin glargine 300 Units · mL-1 provides a more even activity profile and prolonged glycemic control at steady state compared with insulin glargine 100 Units · mL-1. Diabetes care 2015, 38, 637–643, doi:10.2337/dc14-0006. 25150159

[57] Pearson SM; Trujillo JM Conversion from insulin glargine U-100 to insulin glargine U-300 or insulin degludec and the impact on dosage requirements. Ther Adv Endocrinol Metab 2018, 9, 113–121, doi:10.1177/2042018818760962. 29619208 PMC5871063

[58] Havelund S; Plum A; Ribel U; Jonassen I; Vølund A; Markussen J; Kurtzhals P The mechanism of protraction of insulin detemir, a long-acting, acylated analog of human insulin. Pharm Res 2004, 21, 1498–1504, doi:10.1023/b:pham.0000036926.54824.37. 15359587

[59] Klein O; Lynge J; Endahl L; Damholt B; Nosek L; Heise T Albumin-bound basal insulin analogues (insulin detemir and NN344): comparable time-action profiles but less variability than insulin glargine in type 2 diabetes. Diabetes, obesity & metabolism 2007, 9, 290–299, doi:10.1111/j.1463-1326.2006.00685.x. 17391154

[60] Degludec Prescribing Information. Availabe online: https://www.accessdata.fda.gov/drugsatfda_docs/label/2015/203314lbl.pdf (accessed on June 27).

[61] Jonassen I; Havelund S; Hoeg-Jensen T; Steensgaard DB; Wahlund PO; Ribel U Design of the novel protraction mechanism of insulin degludec, an ultra-long-acting basal insulin. Pharm Res 2012, 29, 2104–2114, doi:10.1007/s11095-012-0739-z. 22485010 PMC3399081

[62] Lane W; Bailey TS; Gerety G; Gumprecht J; Philis-Tsimikas A; Hansen CT; Nielsen TSS; Warren M Effect of Insulin Degludec vs Insulin Glargine U100 on Hypoglycemia in Patients With Type 1 Diabetes: The SWITCH 1 Randomized Clinical Trial. Jama 2017, 318, 33–44, doi:10.1001/jama.2017.7115. 28672316 PMC5817477

[63] Holmes RS; Crabtree E; McDonagh MS Comparative effectiveness and harms of long-acting insulins for type 1 and type 2 diabetes: A systematic review and meta-analysis. Diabetes, obesity & metabolism 2019, 21, 984–992, doi:10.1111/dom.13614. 30552792

[64] Thalange N; Deeb L; Iotova V; Kawamura T; Klingensmith G; Philotheou A; Silverstein J; Tumini S; Ocampo Francisco AM; Kinduryte O, Insulin degludec in combination with bolus insulin aspart is safe and effective in children and adolescents with type 1 diabetes. Pediatric diabetes 2015, 16, 164–176, doi:10.1111/pedi.12263. 25683037 PMC4413367

[65] Korsatko S; Deller S; Koehler G; Mader JK; Neubauer K; Adrian CL; Thomsen H; Haahr H; Pieber TR A comparison of the steady-state pharmacokinetic and pharmacodynamic profiles of 100 and 200 U/mL formulations of ultra-long-acting insulin degludec. Clinical drug investigation 2013, 33, 515–521, doi:10.1007/s40261-013-0096-7. 23749405

[66] Cobry E; McFann K; Messer L; Gage V; VanderWel B; Horton L; Chase HP Timing of meal insulin boluses to achieve optimal postprandial glycemic control in patients with type 1 diabetes. Diabetes technology & therapeutics 2010, 12, 173–177, doi:10.1089/dia.2009.0112. 20151766

[67] Evans M; Ceriello A; Danne T; De Block C; DeVries JH; Lind M; Mathieu C; Nørgaard K; Renard E; Wilmot EG Use of fast-acting insulin aspart in insulin pump therapy in clinical practice. Diabetes, obesity & metabolism 2019, 21, 2039–2047, doi:10.1111/dom.13798. PMC6773364 31144428

[68] Bode BW; Iotova V; Kovarenko M; Laffel LM; Rao PV; Deenadayalan S; Ekelund M; Larsen SF; Danne T Efficacy and Safety of Fast-Acting Insulin Aspart Compared With Insulin Aspart, Both in Combination With Insulin Degludec, in Children and Adolescents With Type 1 Diabetes: The onset 7 Trial. Diabetes care 2019, 42, 1255–1262, doi:10.2337/dc19-0009. 31076415 PMC6973646

[69] Fiasp announcement Availabe online: https://www.ajmc.com/view/fda-approves-fiasp-for-children-with-diabetes (accessed on June 27).

[70] Linnebjerg H; Zhang Q; LaBell E; Dellva MA; Coutant DE; Hövelmann U; Plum-Mörschel L; Herbrand T; Leohr J Pharmacokinetics and Glucodynamics of Ultra Rapid Lispro (URLi) versus Humalog(^®^) (Lispro) in Younger Adults and Elderly Patients with Type 1 Diabetes Mellitus: A Randomised Controlled Trial. Clinical pharmacokinetics 2020, 59, 1589–1599, doi:10.1007/s40262-020-00903-0. 32468447 PMC7716921

[71] Ultra Rapid Lispro Approval. Availabe online: https://investor.lilly.com/news-releases/news-release-details/fda-approves-lyumjevtm-insulin-lispro-aabc-injection-lillys-new (accessed on June 27).

[72] Bode BW; Garg SK; Norwood P; Morales C; Hardy T; Liu R; Ignaut D Compatibility and Safety of Ultra Rapid Lispro with Continuous Subcutaneous Insulin Infusion in Patients with Type 1 Diabetes: PRONTO-Pump Study. Diabetes technology & therapeutics 2021, 23, 41–50, doi:10.1089/dia.2020.0224. 32640842

[73] Lasalvia P; Barahona-Correa JE; Romero-Alvernia DM; Gil-Tamayo S; Castañeda-Cardona C; Bayona JG; Triana JJ; Laserna AF; Mejía-Torres M; Restrepo-Jimenez P, Pen Devices for Insulin Self-Administration Compared With Needle and Vial: Systematic Review of the Literature and Meta-Analysis. Journal of diabetes science and technology 2016, 10, 959–966, doi:10.1177/1932296816633721. 26920639 PMC4928229

[74] Ignaut DA; Venekamp WJ HumaPen Memoir: a novel insulin-injecting pen with a dose-memory feature. Expert Rev Med Devices 2007, 4, 793–802, doi:10.1586/17434440.4.6.793. 18035945

[75] Medical C InPen User Guide. Availabe online: https://www.companionmedical.com/guides/inpen-user-guide.pdf (accessed on June 22).

[76] Kampmann U; Madsen LR; Bjerg L; Witte DR; Hasselstrøm K; Østergård T; Alstrup K; Møller MK; Dylmer D; Hansen KW Prevalence and geographical distribution of insulin pump therapy in the Central Denmark Region and its association with metabolic parameters. Diabetes research and clinical practice 2018, 141, 148–155, doi:10.1016/j.diabres.2018.04.042. 29733870

[77] Gajewska KA; Bennett K; Biesma R; Sreenan S Low uptake of continuous subcutaneous insulin infusion therapy in people with type 1 diabetes in Ireland: a retrospective cross-sectional study. BMC endocrine disorders 2020, 20, 92, doi:10.1186/s12902-020-00573-w. 32576284 PMC7310521

[78] Foster NC; Beck RW; Miller KM; Clements MA; Rickels MR; DiMeglio LA; Maahs DM; Tamborlane WV; Bergenstal R; Smith E, State of Type 1 Diabetes Management and Outcomes from the T1D Exchange in 2016-2018. Diabetes technology & therapeutics 2019, 21, 66–72, doi:10.1089/dia.2018.0384. 30657336 PMC7061293

[79] Sora ND; Shashpal F; Bond EA; Jenkins AJ Insulin Pumps: Review of Technological Advancement in Diabetes Management. The American journal of the medical sciences 2019, 358, 326–331, doi:10.1016/j.amjms.2019.08.008. 31655714

[80] Alsaleh FM; Smith FJ; Keady S; Taylor KM Insulin pumps: from inception to the present and toward the future. J Clin Pharm Ther 2010, 35, 127–138, doi:10.1111/j.1365-2710.2009.01048.x. 20456732

[81] Shashaj B; Busetto E; Sulli N Benefits of a bolus calculator in pre- and postprandial glycaemic control and meal flexibility of paediatric patients using continuous subcutaneous insulin infusion (CSII). Diabetic medicine : a journal of the British Diabetic Association 2008, 25, 1036–1042, doi:10.1111/j.1464-5491.2008.02549.x. 18937673

[82] Zisser H; Wagner R; Pleus S; Haug C; Jendrike N; Parkin C; Schweitzer M; Freckmann G Clinical performance of three bolus calculators in subjects with type 1 diabetes mellitus: a head-to-head-to-head comparison. Diabetes technology & therapeutics 2010, 12, 955–961, doi:10.1089/dia.2010.0064. 21128842

[83] Tandem. Control IQ User Guide. Availabe online: https://www.tandemdiabetes.com/docs/default-source/product-documents/t-slim-x2-insulin-pump/aw-1005628_c_user-guide-tslim-x2-control-iq-7-4-mgdl-artwork.pdf?sfvrsn=18a507d7_140 (accessed on June 23).

[84] Medtronic. Minimed 670G User Guide. Availabe online: https://www.medtronicdiabetes.com/download-library/minimed-670g-system (accessed on June 23).

[85] OmniPod User Guide. Availabe online: https://www.omnipod.com/sites/default/files/2021-04/Omnipod-DASH_User-Guide_English.pdf (accessed on June 28).

[86] Medtronic MiniMed640G User Guide. Availabe online: https://hcp.medtronic-diabetes.com.au/sites/default/files/minimed-640g-system-user-guide-mmol.pdf (accessed on June 28).

[87] Siebenhofer A; Jeitler K; Berghold A; Horvath K; Pieber TR Severe hypoglycaemia and glycaemic control in Type 1 diabetes: meta-analysis of multiple daily insulin injections compared with continuous subcutaneous insulin infusion. Diabetic medicine : a journal of the British Diabetic Association 2009, 26, 311–312; author reply 312-313, doi:10.1111/j.1464-5491.2009.02668.x. 19317829

[88] Haynes E; Ley M; Talbot P; Dunbar M; Cummings E Insulin Pump Therapy Improves Quality of Life of Young Patients With Type 1 Diabetes Enrolled in a Government-Funded Insulin Pump Program: A Qualitative Study. Can J Diabetes 2021, 45, 395–402, doi:10.1016/j.jcjd.2020.08.101. 33109446

[89] Al Shaikh A; Al Zahrani AM; Qari YH; AbuAlnasr AA; Alhawsawi WK; Alshehri KA; AlShaikh SA Quality of Life in Children With Diabetes Treated With Insulin Pump Compared With Multiple Daily Injections in Tertiary Care Center. Clinical medicine insights. Endocrinology and diabetes 2020, 13, 1179551420959077, doi:10.1177/1179551420959077. 33088186 PMC7545787

[90] Heller S; White D; Lee E; Lawton J; Pollard D; Waugh N; Amiel S; Barnard K; Beckwith A; Brennan A, A cluster randomised trial, cost-effectiveness analysis and psychosocial evaluation of insulin pump therapy compared with multiple injections during flexible intensive insulin therapy for type 1 diabetes: the REPOSE Trial. Health technology assessment (Winchester, England) 2017, 21, 1–278, doi:10.3310/hta21200. PMC5421095 28440211

[91] Pańkowska E; Szypowska A; Lipka M; Szpotańska M; Błazik M; Groele L Application of novel dual wave meal bolus and its impact on glycated hemoglobin A1c level in children with type 1 diabetes. Pediatric diabetes 2009, 10, 298–303, doi:10.1111/j.1399-5448.2008.00471.x. 19175902

[92] Dos Santos TJ; Donado Campos JM; Argente J; Rodríguez-Artalejo F Effectiveness and equity of continuous subcutaneous insulin infusions in pediatric type 1 diabetes: A systematic review and meta-analysis of the literature. Diabetes research and clinical practice 2021, 172, 108643, doi:10.1016/j.diabres.2020.108643. 33359572

[93] Monami M; Lamanna C; Marchionni N; Mannucci E Continuous subcutaneous insulin infusion versus multiple daily insulin injections in type 1 diabetes: a meta-analysis. Acta diabetologica 2010, 47 Suppl 1, 77–81, doi:10.1007/s00592-009-0132-5. 19504039

[94] Zhang Y; Li S; Zou Y; Wu X; Bi Y; Zhang L; Yuan Y; Gong W; Hayter M Fear of hypoglycemia in patients with type 1 and 2 diabetes: a systematic review. J Clin Nurs 2020, 10.1111/jocn.15538, doi:10.1111/jocn.15538. 33091198

[95] Barnard K; Thomas S; Royle P; Noyes K; Waugh N Fear of hypoglycaemia in parents of young children with type 1 diabetes: a systematic review. BMC pediatrics 2010, 10, 50, doi:10.1186/1471-2431-10-50. 20633252 PMC2912881

[96] Schmidt S; Andersen Nexø M; Norgaard O; Willaing I; Pedersen-Bjergaard U; Skinner TC; Nørgaard K Psychosocial factors associated with HbA(1c) in adults with insulin pump-treated type 1 diabetes: a systematic review. Diabetic medicine : a journal of the British Diabetic Association 2020, 37, 1454–1462, doi:10.1111/dme.14347. 32579748

[97] Medtronic MiniMed Veo User Guide. Availabe online: https://www.medtronic-diabetes.com.au/sites/default/files/veo-step-by-step-guide-fa3-view.pdf (accessed on June 28).

[98] Agrawal P; Welsh JB; Kannard B; Askari S; Yang Q; Kaufman FR Usage and effectiveness of the low glucose suspend feature of the Medtronic Paradigm Veo insulin pump. Journal of diabetes science and technology 2011, 5, 1137–1141, doi:10.1177/193229681100500514. 22027306 PMC3208869

[99] Choudhary P; Shin J; Wang Y; Evans ML; Hammond PJ; Kerr D; Shaw JA; Pickup JC; Amiel SA Insulin pump therapy with automated insulin suspension in response to hypoglycemia: reduction in nocturnal hypoglycemia in those at greatest risk. Diabetes care 2011, 34, 2023–2025, doi:10.2337/dc10-2411. 21868778 PMC3161284

[100] Basal IQ User Guide. Availabe online: https://www.tandemdiabetes.com/docs/default-source/product-documents/t-slim-x2-insulin-pump/aw-1006684_c-user-guide-tslim-x2-basal-iq-6-4-mmoll-artwork-web.pdf?sfvrsn=eeb230d7_139 (accessed on June 28).

[101] Medtronic 640G User Guide. Availabe online: https://hcp.medtronic-diabetes.com.au/sites/default/files/minimed-640g-system-user-guide-mmol.pdf (accessed on June 28).

[102] Beato-Víbora PI; Gil-Poch E; Galán-Bueno L; Lázaro-Martín L; Arroyo-Díez FJ The Incremental Benefits of the Predictive Low-Glucose Suspend Function Compared to the Low-Glucose Suspend Function as Automation Against Hypoglycemia in Sensor-Augmented Pump Therapy. Journal of diabetes science and technology 2018, 12, 1241–1243, doi:10.1177/1932296818791536. 30058373 PMC6232727

[103] Kovatchev B Automated closed-loop control of diabetes: the artificial pancreas. Bioelectron Med 2018, 4, 14, doi:10.1186/s42234-018-0015-6. 32232090 PMC7098217

[104] Medtronic. Medtronic 770G User guide. Availabe online: https://www.medtronicdiabetes.com/download-library/minimed-770g-system (accessed on June 23).

[105] Garg SK; Weinzimer SA; Tamborlane WV; Buckingham BA; Bode BW; Bailey TS; Brazg RL; Ilany J; Slover RH; Anderson SM, Glucose Outcomes with the In-Home Use of a Hybrid Closed-Loop Insulin Delivery System in Adolescents and Adults with Type 1 Diabetes. Diabetes technology & therapeutics 2017, 19, 155–163, doi:10.1089/dia.2016.0421. 28134564 PMC5359676

[106] Forlenza GP; Pinhas-Hamiel O; Liljenquist DR; Shulman DI; Bailey TS; Bode BW; Wood MA; Buckingham BA; Kaiserman KB; Shin J, Safety Evaluation of the MiniMed 670G System in Children 7-13 Years of Age with Type 1 Diabetes. Diabetes technology & therapeutics 2019, 21, 11–19, doi:10.1089/dia.2018.0264. 30585770 PMC6350071

[107] Collyns OJ; Meier RA; Betts ZL; Chan DSH; Frampton C; Frewen CM; Hewapathirana NM; Jones SD; Roy A; Grosman B, Improved Glycemic Outcomes With Medtronic MiniMed Advanced Hybrid Closed-Loop Delivery: Results From a Randomized Crossover Trial Comparing Automated Insulin Delivery With Predictive Low Glucose Suspend in People With Type 1 Diabetes. Diabetes care 2021, 44, 969–975, doi:10.2337/dc20-2250. 33579715

[108] Bergenstal RM; Nimri R; Beck RW; Criego A; Laffel L; Schatz D; Battelino T; Danne T; Weinzimer SA; Sibayan J, A comparison of two hybrid closed-loop systems in adolescents and young adults with type 1 diabetes (FLAIR): a multicentre, randomised, crossover trial. Lancet 2021, 397, 208–219, doi:10.1016/s0140-6736(20)32514-9. 33453783 PMC9194961

[109] Messer LH; Berget C; Vigers T; Pyle L; Geno C; Wadwa RP; Driscoll KA; Forlenza GP Real world hybrid closed-loop discontinuation: Predictors and perceptions of youth discontinuing the 670G system in the first 6 months. Pediatric diabetes 2020, 21, 319–327, doi:10.1111/pedi.12971. 31885123 PMC7204392

[110] Lal RA; Basina M; Maahs DM; Hood K; Buckingham B; Wilson DM One Year Clinical Experience of the First Commercial Hybrid Closed-Loop. Diabetes care 2019, 10.2337/dc19-0855, doi:10.2337/dc19-0855. PMC6868462 31548247

[111] Brown SA; Kovatchev BP; Raghinaru D; Lum JW; Buckingham BA; Kudva YC; Laffel LM; Levy CJ; Pinsker JE; Wadwa RP, Six-Month Randomized, Multicenter Trial of Closed-Loop Control in Type 1 Diabetes. The New England journal of medicine 2019, 10.1056/NEJMoa1907863, doi:10.1056/NEJMoa1907863. PMC7076915 31618560

[112] Breton MD; Kanapka LG; Beck RW; Ekhlaspour L; Forlenza GP; Cengiz E; Schoelwer M; Ruedy KJ; Jost E; Carria L, A Randomized Trial of Closed-Loop Control in Children with Type 1 Diabetes. The New England journal of medicine 2020, 383, 836–845, doi:10.1056/NEJMoa2004736. 32846062 PMC7920146

[113] Breton MD; Kovatchev BP One Year Real-World Use of the Control-IQ Advanced Hybrid Closed-Loop Technology. Diabetes technology & therapeutics 2021, 10.1089/dia.2021.0097, doi:10.1089/dia.2021.0097. PMC8501470 33784196

[114] Brown SA; Forlenza GP; Bode BW; Pinsker JE; Levy CJ; Criego AB; Hansen DW; Hirsch IB; Carlson AL; Bergenstal RM, Multicenter Trial of a Tubeless, On-Body Automated Insulin Delivery System With Customizable Glycemic Targets in Pediatric and Adult Participants With Type 1 Diabetes. Diabetes care 2021, 10.2337/dc21-0172, doi:10.2337/dc21-0172. PMC8323171 34099518

[115] Sherr JL; Buckingham BA; Forlenza GP; Galderisi A; Ekhlaspour L; Wadwa RP; Carria L; Hsu L; Berget C; Peyser TA, Safety and Performance of the Omnipod Hybrid Closed-Loop System in Adults, Adolescents, and Children with Type 1 Diabetes Over 5 Days Under Free-Living Conditions. Diabetes technology & therapeutics 2020, 22, 174–184, doi:10.1089/dia.2019.0286. 31596130 PMC7047109

[116] Buckingham BA; Forlenza GP; Pinsker JE; Christiansen MP; Wadwa RP; Schneider J; Peyser TA; Dassau E; Lee JB; O’Connor J, Safety and Feasibility of the OmniPod Hybrid Closed-Loop System in Adult, Adolescent, and Pediatric Patients with Type 1 Diabetes Using a Personalized Model Predictive Control Algorithm. Diabetes technology & therapeutics 2018, 20, 257–262, doi:10.1089/dia.2017.0346. 29431513 PMC5910038

[117] Cam APS FX User Guide. Availabe online: https://s3-eu-west-1.amazonaws.com/camdiab.user.manual/user_manual_fx_mmoll_commercial.pdf (accessed on June 29).

[118] Leelarathna L; Choudhary P; Wilmot EG; Lumb A; Street T; Kar P; Ng SM Hybrid closed-loop therapy: Where are we in 2021? Diabetes, obesity & metabolism 2021, 23, 655–660, doi:10.1111/dom.14273. 33269551

[119] Chen NS; Boughton CK; Hartnell S; Fuchs J; Allen JM; Willinska ME; Thankamony A; de Beaufort C; Campbell FM; Fröhlich-Reiterer E, User Engagement With the CamAPS FX Hybrid Closed-Loop App According to Age and User Characteristics. Diabetes care 2021, 10.2337/dc20-2762, doi:10.2337/dc20-2762. PMC8323184 34021021

[120] Thabit H; Tauschmann M; Allen JM; Leelarathna L; Hartnell S; Wilinska ME; Acerini CL; Dellweg S; Benesch C; Heinemann L, Home Use of an Artificial Beta Cell in Type 1 Diabetes. The New England journal of medicine 2015, 373, 2129–2140, doi:10.1056/NEJMoa1509351. 26379095 PMC4697362

[121] Amadou C; Franc S; Benhamou PY; Lablanche S; Huneker E; Charpentier G; Penfornis A Diabeloop DBLG1 Closed-Loop System Enables Patients With Type 1 Diabetes to Significantly Improve Their Glycemic Control in Real-Life Situations Without Serious Adverse Events: 6-Month Follow-up. Diabetes care 2021, 44, 844–846, doi:10.2337/dc20-1809. 33431420

[122] Benhamou PY; Huneker E; Franc S; Doron M; Charpentier G Customization of home closed-loop insulin delivery in adult patients with type 1 diabetes, assisted with structured remote monitoring: the pilot WP7 Diabeloop study. Acta diabetologica 2018, 55, 549–556, doi:10.1007/s00592-018-1123-1. 29520615

[123] Viñals C; Beneyto A; Martín-SanJosé JF; Furió-Novejarque C; Bertachi A; Bondia J; Vehi J; Conget I; Giménez M Artificial Pancreas With Carbohydrate Suggestion Performance for Unannounced and Announced Exercise in Type 1 Diabetes. The Journal of clinical endocrinology and metabolism 2021, 106, 55–63, doi:10.1210/clinem/dgaa562. 32852548

[124] Omer T Empowered citizen ‘health hackers’ who are not waiting. BMC medicine 2016, 14, 118, doi:10.1186/s12916-016-0670-y. 27530970 PMC4988004

[125] Loop and Learn - FreeAPS. Availabe online: https://www.loopnlearn.org/freeapsdoc/ (accessed on June 23).

[126] Kesavadev J; Srinivasan S; Saboo B; Krishna BM; Krishnan G The Do-It-Yourself Artificial Pancreas: A Comprehensive Review. Diabetes Ther 2020, 11, 1217–1235, doi:10.1007/s13300-020-00823-z. 32356245 PMC7261300

[127] OpenAPS outcomes. Availabe online: https://openaps.org/outcomes/ (accessed on June, 23).

[128] Lum J Loop Observational Study - Evaluating Do-It Yourself (DIY) Automated Insulin Delivery. ATTD 2020, Madrid 2020.

[129] Tidepool iAC. Availabe online: https://www.tidepool.org/blog/interoperability-is-the-how-not-the-why (accessed on July 15).

[130] Toffanin C; Kozak M; Sumnik Z; Cobelli C; Petruzelkova L In Silico Trials of an Open-Source Android-Based Artificial Pancreas: A New Paradigm to Test Safety and Efficacy of Do-It-Yourself Systems. Diabetes technology & therapeutics 2019, 10.1089/dia.2019.0375, doi:10.1089/dia.2019.0375. 31769699

[131] Jennings P; Hussain S Do-It-Yourself Artificial Pancreas Systems: A Review of the Emerging Evidence and Insights for Healthcare Professionals. Journal of diabetes science and technology 2020, 14, 868–877, doi:10.1177/1932296819894296. 31847570 PMC7753866

[132] Mack GS Pfizer dumps Exubera. Nature biotechnology 2007, 25, 1331–1332, doi:10.1038/nbt1207-1331. 18066009

[133] Afrezza Prescribing Information. Availabe online: https://www.afrezza.com/pdf/Full-Prescribing-Information.pdf (accessed on June 23).

[134] Gerber RA; Cappelleri JC; Kourides IA; Gelfand RA Treatment satisfaction with inhaled insulin in patients with type 1 diabetes: a randomized controlled trial. Diabetes care 2001, 24, 1556–1559, doi:10.2337/diacare.24.9.1556. 11522698

[135] Royle P; Waugh N; McAuley L; McIntyre L; Thomas S Inhaled insulin in diabetes mellitus. Cochrane Database Syst Rev 2003, 10.1002/14651858.Cd003890, Cd003890, doi:10.1002/14651858.Cd003890. 12917994

[136] Pittas AG; Westcott GP; Balk EM Efficacy, safety, and patient acceptability of Technosphere inhaled insulin for people with diabetes: a systematic review and meta-analysis. The lancet. Diabetes & endocrinology 2015, 3, 886–894, doi:10.1016/s2213-8587(15)00280-6. 26341170

[137] Galderisi A; Cohen N; Calhoun P; Kraemer K; Breton M; Weinzimer S; Cengiz E Effect of Afrezza on Glucose Dynamics During HCL Treatment. Diabetes care 2020, 43, 2146–2152, doi:10.2337/dc20-0091. 32661108 PMC7440894

[138] Santos LL; Lima FJC; Sousa-Rodrigues CF; Barbosa FT Use of SGLT-2 inhibitors in the treatment of type 2 diabetes mellitus. Revista da Associacao Medica Brasileira (1992) 2017, 63, 636–641, doi:10.1590/1806-9282.63.07.636. 28977090

[139] Buse JB; Garg SK; Rosenstock J; Bailey TS; Banks P; Bode BW; Danne T; Kushner JA; Lane WS; Lapuerta P, Sotagliflozin in Combination With Optimized Insulin Therapy in Adults With Type 1 Diabetes: The North American inTandem1 Study. Diabetes care 2018, 41, 1970–1980, doi:10.2337/dc18-0343. 29937430 PMC6105319

[140] Dandona P; Mathieu C; Phillip M; Hansen L; Tschope D; Thoren F; Xu J; Langkilde AM Efficacy and Safety of Dapagliflozin in Patients With Inadequately Controlled Type 1 Diabetes: The DEPICT-1 52-Week Study. Diabetes care 2018, 41, 2552–2559, doi:10.2337/dc18-1087. 30352894

[141] Rosenstock J; Marquard J; Laffel LM; Neubacher D; Kaspers S; Cherney DZ; Zinman B; Skyler JS; George J; Soleymanlou N, Empagliflozin as Adjunctive to Insulin Therapy in Type 1 Diabetes: The EASE Trials. Diabetes care 2018, 41, 2560–2569, doi:10.2337/dc18-1749. 30287422

[142] Yu X; Zhang S; Zhang L Newer Perspectives of Mechanisms for Euglycemic Diabetic Ketoacidosis. International journal of endocrinology 2018, 2018, 7074868, doi:10.1155/2018/7074868. 30369948 PMC6189664

[143] Lu J; Tang L; Meng H; Zhao J; Liang Y Effects of sodium-glucose cotransporter (SGLT) inhibitors in addition to insulin therapy on glucose control and safety outcomes in adults with type 1 diabetes: A meta-analysis of randomized controlled trials. Diabetes/metabolism research and reviews 2019, 35, e3169, doi:10.1002/dmrr.3169. 30974510

[144] Teymourian H; Moonla C; Tehrani F; Vargas E; Aghavali R; Barfidokht A; Tangkuaram T; Mercier PP; Dassau E; Wang J Microneedle-Based Detection of Ketone Bodies along with Glucose and Lactate: Toward Real-Time Continuous Interstitial Fluid Monitoring of Diabetic Ketosis and Ketoacidosis. Anal Chem 2020, 92, 2291–2300, doi:10.1021/acs.analchem.9b05109. 31874029

[145] Flores M; Amir M; Ahmed R; Alashi S; Li M; Wang X; Lansang MC; Al-Jaghbeer MJ Causes of diabetic ketoacidosis among adults with type 1 diabetes mellitus: insulin pump users and non-users. BMJ Open Diabetes Res Care 2020, 8, doi:10.1136/bmjdrc-2020-001329. PMC7737023 33318067

[146] Miller E; Doshi A; Grøn R; Jódar E; Őrsy P; Ranthe MF; Sugimoto D; Tentolouris N; Viljoen A; Billings LK IDegLira improves patient-reported outcomes while using a simple regimen with fewer injections and dose adjustments compared with basal-bolus therapy. Diabetes, obesity & metabolism 2019, 21, 2643–2650, doi:10.1111/dom.13851. PMC6899651 31385425

[147] Rosenstock J; Bajaj HS; Janež A; Silver R; Begtrup K; Hansen MV; Jia T; Goldenberg R Once-Weekly Insulin for Type 2 Diabetes without Previous Insulin Treatment. The New England journal of medicine 2020, 383, 2107–2116, doi:10.1056/NEJMoa2022474. 32960514

[148] Ozer K; Cooper AM; Ahn LP; Waggonner CR; Blevins TC Fast Acting Insulin Aspart Compared with Insulin Aspart in the Medtronic 670G Hybrid Closed Loop System in Type 1 Diabetes: An Open Label Crossover Study. Diabetes technology & therapeutics 2021, 23, 286–292, doi:10.1089/dia.2020.0500. 33090016 PMC7994433

[149] Grosman B; Wu D; Parikh N; Roy A; Voskanyan G; Kurtz N; Sturis J; Cohen O; Ekelund M; Vigersky R Fast-acting insulin aspart (Fiasp^®^) improves glycemic outcomes when used with MiniMed(TM) 670G hybrid closed-loop system in simulated trials compared to NovoLog^®^. Comput Methods Programs Biomed 2021, 205, 106087, doi:10.1016/j.cmpb.2021.106087. 33873075

[150] Hsu L; Buckingham B; Basina M; Ekhlaspour L; von Eyben R; Wang J; Lal RA Fast-Acting Insulin Aspart Use with the MiniMed(TM) 670G System. Diabetes technology & therapeutics 2021, 23, 1–7, doi:10.1089/dia.2020.0083. 32520594 PMC7864093

[151] Boughton CK; Hartnell S; Thabit H; Poettler T; Herzig D; Wilinska ME; Ashcroft NL; Sibayan J; Cohen N; Calhoun P, Hybrid closed-loop glucose control with faster insulin aspart compared with standard insulin aspart in adults with type 1 diabetes: A double-blind, multicentre, multinational, randomized, crossover study. Diabetes, obesity & metabolism 2021, 23, 1389–1396, doi:10.1111/dom.14355. PMC11497277 33606901

[152] Tsoukas MA; Cohen E; Legault L; von Oettingen JE; Yale JF; Vallis M; Odabassian M; El Fathi A; Rutkowski J; Jafar A, Alleviating carbohydrate counting with a FiASP-plus-pramlintide closed-loop delivery system (artificial pancreas): Feasibility and pilot studies. Diabetes, obesity & metabolism 2021, 10.1111/dom.14447, doi:10.1111/dom.14447. 34047449

[153] OpenAPS. Availabe online: https://openaps.readthedocs.io/en/latest/docs/Customize-Iterate/oref1.html#understanding-unannounced-meals-uam (accessed on June 27).

[154] Pohl R; Li M; Krasner A; De Souza E Development of stable liquid glucagon formulations for use in artificial pancreas. Journal of diabetes science and technology 2015, 9, 8–16, doi:10.1177/1932296814555541. 25352634 PMC4495524

[155] Castle JR; Youssef JE; Branigan D; Newswanger B; Strange P; Cummins M; Shi L; Prestrelski S Comparative Pharmacokinetic/Pharmacodynamic Study of Liquid Stable Glucagon Versus Lyophilized Glucagon in Type 1 Diabetes Subjects. Journal of diabetes science and technology 2016, 10, 1101–1107, doi:10.1177/1932296816653141. 27325390 PMC5032962

[156] Blair HA Dasiglucagon: First Approval. Drugs 2021, 81, 1115–1120, doi:10.1007/s40265-021-01531-z. 34047955

[157] Taleb N; Emami A; Suppere C; Messier V; Legault L; Ladouceur M; Chiasson JL; Haidar A; Rabasa-Lhoret R Efficacy of single-hormone and dual-hormone artificial pancreas during continuous and interval exercise in adult patients with type 1 diabetes: randomised controlled crossover trial. Diabetologia 2016, 59, 2561–2571, doi:10.1007/s00125-016-4107-0. 27704167

[158] Castle JR; El Youssef J; Wilson LM; Reddy R; Resalat N; Branigan D; Ramsey K; Leitschuh J; Rajhbeharrysingh U; Senf B, Randomized Outpatient Trial of Single- and Dual-Hormone Closed-Loop Systems That Adapt to Exercise Using Wearable Sensors. Diabetes care 2018, 41, 1471–1477, doi:10.2337/dc18-0228. 29752345 PMC6014543

[159] Haidar A; Legault L; Messier V; Mitre TM; Leroux C; Rabasa-Lhoret R Comparison of dual-hormone artificial pancreas, single-hormone artificial pancreas, and conventional insulin pump therapy for glycaemic control in patients with type 1 diabetes: an open-label randomised controlled crossover trial. The lancet. Diabetes & endocrinology 2015, 3, 17–26, doi:10.1016/s2213-8587(14)70226-8. 25434967

[160] Russell SJ; Hillard MA; Balliro C; Magyar KL; Selagamsetty R; Sinha M; Grennan K; Mondesir D; Ekhlaspour L; Zheng H, Day and night glycaemic control with a bionic pancreas versus conventional insulin pump therapy in preadolescent children with type 1 diabetes: a randomised crossover trial. The lancet. Diabetes & endocrinology 2016, 4, 233–243, doi:10.1016/s2213-8587(15)00489-1. 26850709 PMC4799495

[161] Castellanos LE; Balliro CA; Sherwood JS; Jafri R; Hillard MA; Greaux E; Selagamsetty R; Zheng H; El-Khatib FH; Damiano ER, Performance of the Insulin-Only iLet Bionic Pancreas and the Bihormonal iLet Using Dasiglucagon in Adults With Type 1 Diabetes in a Home-Use Setting. Diabetes care 2021, 10.2337/dc20-1086, doi:10.2337/dc20-1086. PMC8247518 33906916

[162] Wilson LM; Jacobs PG; Ramsey KL; Resalat N; Reddy R; Branigan D; Leitschuh J; Gabo V; Guillot F; Senf B, Dual-Hormone Closed-Loop System Using a Liquid Stable Glucagon Formulation Versus Insulin-Only Closed-Loop System Compared With a Predictive Low Glucose Suspend System: An Open-Label, Outpatient, Single-Center, Crossover, Randomized Controlled Trial. Diabetes care 2020, 43, 2721–2729, doi:10.2337/dc19-2267. 32907828

[163] El-Khatib FH; Balliro C; Hillard MA; Magyar KL; Ekhlaspour L; Sinha M; Mondesir D; Esmaeili A; Hartigan C; Thompson MJ, Home use of a bihormonal bionic pancreas versus insulin pump therapy in adults with type 1 diabetes: a multicentre randomised crossover trial. Lancet 2017, 389, 369–380, doi:10.1016/s0140-6736(16)32567-3. 28007348 PMC5358809

[164] Peters TM; Haidar A Dual-hormone artificial pancreas: benefits and limitations compared with single-hormone systems. Diabetic medicine : a journal of the British Diabetic Association 2018, 35, 450–459, doi:10.1111/dme.13581. 29337384

[165] Sherr JL; Patel NS; Michaud CI; Palau-Collazo MM; Van Name MA; Tamborlane WV; Cengiz E; Carria LR; Tichy EM; Weinzimer SA Mitigating Meal-Related Glycemic Excursions in an Insulin-Sparing Manner During Closed-Loop Insulin Delivery: The Beneficial Effects of Adjunctive Pramlintide and Liraglutide. Diabetes care 2016, 39, 1127–1134, doi:10.2337/dc16-0089. 27208332 PMC4915555

[166] Mathieu C; Zinman B; Hemmingsson JU; Woo V; Colman P; Christiansen E; Linder M; Bode B Efficacy and Safety of Liraglutide Added to Insulin Treatment in Type 1 Diabetes: The ADJUNCT ONE Treat-To-Target Randomized Trial. Diabetes care 2016, 39, 1702–1710, doi:10.2337/dc16-0691. 27506222

[167] Dejgaard TF; Frandsen CS; Hansen TS; Almdal T; Urhammer S; Pedersen-Bjergaard U; Jensen T; Jensen AK; Holst JJ; Tarnow L, Efficacy and safety of liraglutide for overweight adult patients with type 1 diabetes and insufficient glycaemic control (Lira-1): a randomised, double-blind, placebo-controlled trial. The lancet. Diabetes & endocrinology 2016, 4, 221–232, doi:10.1016/s2213-8587(15)00436-2. 26656289

[168] Riddell MC; Gallen IW; Smart CE; Taplin CE; Adolfsson P; Lumb AN; Kowalski A; Rabasa-Lhoret R; McCrimmon RJ; Hume C, Exercise management in type 1 diabetes: a consensus statement. The lancet. Diabetes & endocrinology 2017, 5, 377–390, doi:10.1016/s2213-8587(17)30014-1. 28126459

[169] Ploug T; Galbo H; Richter EA Increased muscle glucose uptake during contractions: no need for insulin. The American journal of physiology 1984, 247, E726–731, doi:10.1152/ajpendo.1984.247.6.E726. 6391198

[170] Hawley JA; Lessard SJ Exercise training-induced improvements in insulin action. Acta Physiol (Oxf) 2008, 192, 127–135, doi:10.1111/j.1748-1716.2007.01783.x. 18171435

[171] Jacobs PG; El Youssef J; Reddy R; Resalat N; Branigan D; Condon J; Preiser N; Ramsey K; Jones M; Edwards C, Randomized trial of a dual-hormone artificial pancreas with dosing adjustment during exercise compared with no adjustment and sensor-augmented pump therapy. Diabetes, obesity & metabolism 2016, 18, 1110–1119, doi:10.1111/dom.12707. PMC5056819 27333970

[172] Resalat N; El Youssef J; Reddy R; Jacobs PG Design of a dual-hormone model predictive control for artificial pancreas with exercise model. Annu Int Conf IEEE Eng Med Biol Soc 2016, 2016, 2270–2273, doi:10.1109/embc.2016.7591182. 28324962 PMC5858942

[173] Jayawardene DC; McAuley SA; Horsburgh JC; Gerche A; Jenkins AJ; Ward GM; MacIsaac RJ; Roberts TJ; Grosman B; Kurtz N, Closed-Loop Insulin Delivery for Adults with Type 1 Diabetes Undertaking High-Intensity Interval Exercise Versus Moderate-Intensity Exercise: A Randomized, Crossover Study. Diabetes technology & therapeutics 2017, 19, 340–348, doi:10.1089/dia.2016.0461. 28574723

[174] Breton MD; Brown SA; Karvetski CH; Kollar L; Topchyan KA; Anderson SM; Kovatchev BP Adding heart rate signal to a control-to-range artificial pancreas system improves the protection against hypoglycemia during exercise in type 1 diabetes. Diabetes technology & therapeutics 2014, 16, 506–511, doi:10.1089/dia.2013.0333. 24702135 PMC4116126

[175] Lee MH; Paldus B; Krishnamurthy B; McAuley SA; Shah R; Jenkins AJ; O’Neal DN The Clinical Case for the Integration of a Ketone Sensor as Part of a Closed Loop Insulin Pump System. Journal of diabetes science and technology 2019, 13, 967–973, doi:10.1177/1932296818822986. 30628470 PMC6955455

[176] Alva S; Castorino K; Cho H; Ou J Feasibility of Continuous Ketone Monitoring in Subcutaneous Tissue using a Ketone Sensor. Journal of diabetes science and technology 2021, 10.1177/19322968211008185, 19322968211008185, doi:10.1177/19322968211008185. PMC8252149 33832353

[177] O’Connor MR; Carlin K; Coker T; Zierler B; Pihoker C Disparities in Insulin Pump Therapy Persist in Youth With Type 1 Diabetes Despite Rising Overall Pump Use Rates. Journal of pediatric nursing 2019, 44, 16–21, doi:10.1016/j.pedn.2018.10.005. 30581163 PMC10602396

[178] Renard E; Tubiana-Rufi N; Bonnemaison-Gilbert E; Coutant R; Dalla-Vale F; Farret A; Poidvin A; Bouhours-Nouet N; Abettan C; Storey-London C, Closed-loop driven by control-to-range algorithm outperforms threshold-low-glucose-suspend insulin delivery on glucose control albeit not on nocturnal hypoglycaemia in prepubertal patients with type 1 diabetes in a supervised hotel setting. Diabetes, obesity & metabolism 2019, 21, 183–187, doi:10.1111/dom.13482. 30047223

[179] American Diabetes, A. Help with Insulin is a phone call away. Availabe online: https://insulinhelp.org (accessed on June 28).

[180] Shafie AA; Ng CH; Tan YP; Chaiyakunapruk N Systematic Review of the Cost Effectiveness of Insulin Analogues in Type 1 and Type 2 Diabetes Mellitus. Pharmacoeconomics 2017, 35, 141–162, doi:10.1007/s40273-016-0456-2. 27752998

[181] Walker AF; Hood KK; Gurka MJ; Filipp SL; Anez-Zabala C; Cuttriss N; Haller MJ; Roque X; Naranjo D; Aulisio G, Barriers to Technology Use and Endocrinology Care for Underserved Communities With Type 1 Diabetes. Diabetes care 2021, 10.2337/dc20-2753, doi:10.2337/dc20-2753. PMC8323174 34001535

[182] Sumnik Z; Szypowska A; Iotova V; Bratina N; Cherubini V; Forsander G; Jali S; Raposo JF; Stipančic G; Vazeou A, Persistent heterogeneity in diabetes technology reimbursement for children with type 1 diabetes: The SWEET perspective. Pediatric diabetes 2019, 20, 434–443, doi:10.1111/pedi.12833. 30773756

[183] Lipman TH; Smith JA; Patil O; Willi SM; Hawkes CP Racial disparities in treatment and outcomes of children with type 1 diabetes. Pediatric diabetes 2021, 22, 241–248, doi:10.1111/pedi.13139. 33871154

[184] Fantasia KL; Wirunsawanya K; Lee C; Rizo I Racial Disparities in Diabetes Technology Use and Outcomes in Type 1 Diabetes in a Safety-Net Hospital. Journal of diabetes science and technology 2021, 10.1177/1932296821995810, 1932296821995810, doi:10.1177/1932296821995810. PMC8442173 33719610

[185] Walker AF; Haller MJ; Gurka MJ; Morris HL; Bruggeman B; Miller K; Foster N; Anez Zabala C; Schatz DA Addressing health disparities in type 1 diabetes through peer mentorship. Pediatric diabetes 2020, 21, 120–127, doi:10.1111/pedi.12935. 31617648

[186] Peters AL; Garg SK The Silver Lining to COVID-19: Avoiding Diabetic Ketoacidosis Admissions with Telehealth. Diabetes technology & therapeutics 2020, 22, 449–453, doi:10.1089/dia.2020.0187. 32383989

